# MicroRNA-93-5p regulates odontogenic differentiation and dentin formation via KDM6B

**DOI:** 10.1186/s12967-024-04862-z

**Published:** 2024-01-13

**Authors:** Si Wu, Xin Xu, Shiqi Gao, Sibei Huo, Mian Wan, Xin Zhou, Xuedong Zhou, Liwei Zheng, Yachuan Zhou

**Affiliations:** 1 State Key Laboratory of Oral Diseases & National Clinical Research Center for Oral Diseases & Department of Cariology and Endodontics, West China Hospital of Stomatology, Sichuan University, No. 14, Section 3, Renmin South Road, Chengdu, 610041 Sichuan China; 2 State Key Laboratory of Oral Diseases & National Clinical Research Center for Oral Diseases & Department of Pediatric Dentistry, West China Hospital of Stomatology, Sichuan University, No. 14, Section 3, Renmin South Road, Chengdu, 610041 Sichuan China

**Keywords:** MicroRNA-93-5p, KDM6B, Dental pulp stem cells, Tertiary dentin, Pulpotomy

## Abstract

**Background:**

Epigenetic factors influence the odontogenic differentiation of dental pulp stem cells and play indispensable roles during tooth development. Some microRNAs can epigenetically regulate other epigenetic factors like DNA methyltransferases and histone modification enzymes, functioning as epigenetic-microRNAs. In our previous study, microarray analysis suggested microRNA-93-5p (miR-93-5p) was differentially expressed during the bell stage in human tooth germ. Prediction tools indicated that miR-93-5p may target lysine-specific demethylase 6B (KDM6B). Therefore, we explored the role of miR-93-5p as an epi-miRNA in tooth development and further investigated the underlying mechanisms of miR-93-5p in regulating odontogenic differentiation and dentin formation.

**Methods:**

The expression pattern of miR-93-5p and KDM6B of dental pulp stem cells (DPSCs) was examined during tooth development and odontogenic differentiation. Dual luciferase reporter and ChIP-qPCR assay were used to validate the target and downstream regulatory genes of miR-93-5p in human DPSCs (hDPSCs). Histological analyses and qPCR assays were conducted for investigating the effects of miR-93-5p mimic and inhibitor on odontogenic differentiation of hDPSCs. A pulpotomy rat model was further established, microCT and histological analyses were performed to explore the effects of KDM6B-overexpression and miR-93-5p inhibition on the formation of tertiary dentin.

**Results:**

The expression level of miR-93-5p decreased as odontoblast differentiated, in parallel with elevated expression of histone demethylase KDM6B. In hDPSCs, miR-93-5p overexpression inhibited the odontogenic differentiation and vice versa. MiR-93-5p targeted 3′ untranslated region (UTR) of KDM6B, thereby inhibiting its protein translation. Furthermore, KDM6B bound the promoter region of *BMP2* to demethylate H3K27me3 marks and thus upregulated BMP2 transcription. In the rat pulpotomy model, KDM6B-overexpression or miR-93-5p inhibition suppressed H3K27me3 level in DPSCs and consequently promoted the formation of tertiary dentin.

**Conclusions:**

MiR-93-5p targets epigenetic regulator KDM6B and regulates H3K27me3 marks on *BMP2* promoters, thus modulating the odontogenic differentiation of DPSCs and dentin formation.

**Supplementary Information:**

The online version contains supplementary material available at 10.1186/s12967-024-04862-z.

## Background

Tooth development is a time- and space-specific process including the initiation, bud, cap, and bell stages. In the past few decades, the molecular pathways and regulating mechanisms underlying tooth morphogenesis have been widely explored [1]. In tooth germ, the intimate interactivities between dental epithelium and mesenchyme tissues are sequentially controlled by multiple cytokines/signaling molecules, including bone morphogenetic proteins (BMPs), Wnt, and Shh [2–4]. During the bell stage, the inner enamel epithelial cells differentiate into enamel-secreting ameloblasts, while the adjacent dental papilla mesenchymal cells polarize and differentiate into odontoblasts to secrete dentin matrix [5]. Subsequently, the dental papilla mesenchyme encompassed by accumulative dentin matrix forms dental pulp. The outside enamel and dentin are hard component of tooth, protecting the dental pulp tissues.

The dentin and dental pulp are together called dental-pulp complex because of their close relationships in biological development and physiological structure. The dental-pulp complex is crucial for the life of tooth, not only because of the commonly physiologic functions of pulp, but also the regulating effects on pulp homeostasis. After severe pulp injury, odontoblasts differentiated from dental pulp stem cells (DPSCs) form reparative dentinogenesis.

Human dental pulp stem cells (hDPSCs) are isolated from adult dental pulp tissues and positive for mesenchymal stem cells markers [6]. As multipotent progenitors, hDPSCs are able to self-renewal and differentiate into dentin-forming odontoblasts [7]. Multiple growth factors and complex molecular signal pathways are related to odontogenic differentiation of hDPSCs and dentinogenesis, including BMPs, insulin-like growth factor, vascular endothelial growth factor and platelet-derived growth factor [8]. Reasonably, DPSCs are considered to be a promising and suitable source for in vivo and in vitro studies of tertiary dentin formation and dental pulp regeneration [9–12].

Numerous studies have explored biomolecular capping materials to promote the repair of injured pulp tissue [13, 14]. Among these, microRNAs (miRNAs) are promising molecules due to their epigenetic regulatory role in multiple biological processes like osteo/odontogenic differentiation [15–20]. By binding to the 3′UTR of messenger RNAs, some microRNAs are proved to influence the odontogenic differentiation of DPSCs in post-transcriptional level by negatively regulate the target genes like krüpple-like factor 4, bone morphogenetic protein receptor type II, osterix and glycoprotein non-metastatic melanomal protein B [16, 21–23]. Furthermore, studies have revealed that some microRNAs can epigenetically regulate other epigenetic factors like DNA methyltransferases and histone modification enzymes, functioning as epigenetic-microRNAs (epi-miRNAs) [24]. After odontogenic induction of hDPSCs, the differentially expressed miRNAs have been analyzed [20]. However, whether these miRNAs can work as epi-miRNA and the underlying regulation pattern still need to be explored.

In addition to the epigenetic regulation by miRNA, posttranslational modifications of histone proteins are also closely associated with odontoblast differentiation and tooth development [25–28]. In bell stage of mice tooth germ, the lysine 27 trimethylation on histone 3 (H3K27me3) marks of dental papilla showed a spatiotemporal pattern and decreased from early to late bell stage. During the odontogenic differentiation process of human dental papilla cells, the dynamic levels of H3K27me3 marks accompanied by the elevated trend of specific histone demethylase KDM6B [29]. Besides, the KDM6B was found to remove the H3K27me3 marks from the promoter region of *BMP2* to promote odontogenic differentiation [30, 31]. These modifications at histone level represent a complicated and dynamic process. Studies have analyzed the miRNAs that differentially expressed during tooth development and odontogenic differentiation of DPSCs, however, if these miRNAs could interplay with these epigenetic modifiers and further influence the tooth development in histone modification levels still need further studies.

Our previous study analyzed the miRNAs in human tooth germs during bell stage and found significantly varied expression of miR-93-5p [32]. As a member of miR-106b-25 cluster, miR-93-5p had been proved to play a functional role in osteoarthritis by affecting anti-inflammation and associate to the recovery of sepsis related acute kidney injury by targeting to KDM6B [19, 33–36]. In present study, the potential functions of miR-93-5p working as an epi-miRNA in dentin formation of tooth development and odontogenic differentiation were investigated.

## Methods

### Oligonucleotide transfection

HDPSCs were cultured as reported method [37] and approval from the Medical Ethics Committee of West China Hospital of Stomatology, Sichuan University (WCHSIRB-CT-2021-243). Hsa-miR-93-5p mimic (miR-10000093-1-5, Ribobio), mimic NC (miR-1N0000001-1-5, Ribobio), hsa-miR-93-5p inhibitor (miR-20000093-1-5, Ribobio) and inhibitor NC (miR-2N0000001-1-5, Ribobio) were synthesized. Lipofectamine 3000 reagents (Invitrogen) was used for oligonucleotide transfection. The final concentrations in the transfection system were 50 nM. The mimic/inhibitor NC were controls.

### Dual-luciferase reporter assay

Synthetic KDM6B-WT-3′UTR (wild type) and KDM6B-MUT-3′UTR (mutant) gene fragment were cloned into pGLO vectors separately, and then co-transfected with 293T cells and miR-93-5p/NC mimic in virtue of Lipofectamine 3000 Reagents. Cell suspension was collected and luciferase activities of samples were detected.

### qRT-PCR and western blotting

Total RNAs including miRNAs in hDPSCs were prepared by RNeasy Plus Mini Kit (74134, Qiagen) and mice tooth tissues were prepared by RNeasy Plus Micro Kit (74034, Qiagen). After reverse transcription, samples were processed for quantitative polymerase chain reaction (qPCR). Tables 1 and 2 listed the primers.

**Table 1:** The primer sequences for qRT-PCR of mice tooth tissues

Primers	Sequences	
*Gapdh*	F	ACTGAGGACCAGGTTGTC
	R	TGCTGTAGCCGTATTCATTG
*Ck14*	F	ACATTAAAATGCCAAGCCCCA
	R	TGATCCCGCATCTCGTTCAG
*Vimentin*	F	TACATCGACAAGGTGCGCTT
	R	CACGCTTTCATACTGCTGGC
*Osx*	F	AGTGGGAACAAGAGTGAGCTG
	R	TAGTGAGCTTCTTCCTGGGT
*Col-1α*	F	GCGCTAAAGGTGCCAATG
	R	AGCACCAGGTTCACCACTG
*Kdm6b*	F	CAATCCCCGCAGAGCTTACC
	R	TTCTACTGGAGGTGGTGCATT

**Table 2:** The primer sequences for qRT-PCR of hDPSCs

Primers	Sequences	
*GAPDH*	F	CGGACCAATACGACCAAATCCG
	R	AGCCACATCGCTCAGACACC
*OCN*	F	ATTGTGGCTCACCCTCCATC
	R	CCAGCCTCCAGCACTGTTTA
*OSX*	F	TCTGCGGGACTCAACAACTC
	R	TAGCATAGCCTGAGGTGGGT
*ALP*	F	CTATCCTGGCTCCGTGCTCC
	R	GTTAACTGATGTTCCAATCCTGCG
*COL-1α*	F	AGGGACACAGAGGTTTCAGT
	R	AGCACCATCATTTCCACGAG
*BMP-2*	F	GTCAACTCGATGCTGTACCTTGACG
	R	CAACCCTCCACAACCATGTCC
*KDM6A*	F	GGCAGTGGAACGGTACGAAT
	R	TCCTGCAGCAATGAGAGCTT
*KDM6B*	F	CCTGAGGGTGAGCAACTCC
	R	GGGGGTGAAGGTCTGTGTTTT
*SUZ12*	F	CCTGGAAGTCCTGCTTGTGA
	R	ACTGGAAACTGCAAGGGACG
*EED*	F	AAATCCACCCTGGGATTCGG
	R	TGGCGAATGGAAAGTACCCG
*EZH2*	F	GGGACTCAGAAGGCAGTGG
	R	TGCACAGGCTGTATCCTTCG

Total proteins were extracted according to the protocol (KeyGEN). The primary antibodies were β-actin (1:1000, GB11001, Servicebio), Histone 3 (1:1000, GB11102, Servicebio), H3K27me3 (1:700, 6002, Abcam) and KDM6B (1:700, ab169197, Abcam). Secondary antibodies of goat anti-rabbit/mouse IgG-HRP (1:5000, L3012-2/L3032-2, Signal way Antibody) were used.

### Alizarin Red S and ALP staining

Base medium (NC) and odontogenic induction medium (OM) for cells culture were compounded as previous description [38]. After odontogenic induction for 3, 7 and 14 days, hDPSCs were fixed and dyed by Alkaline Phosphatase (ALP) Assay Kit (Biyotime). Besides, mineral nodules were stained and observed by Alizarin Red S solution (Solarbio). Images were acquired with inverted light microscopy (Olympus, Japan).

### Chromatin immunoprecipitation (ChIP) assays

Cells were harvested after transfected with miR-93-5p mimic and odontogenic induction for 7 days. Enzymatically processed chromatin was obtained by EZ-Zyme Chromatin Prep Kit (17-375, Millipore). EZ-Magna ChIP HiSens kit (17-10461, Millipore) and antibodies of rabbit anti-H3K27me3 (9733, Cell Signaling Technology), rabbit anti-KDM6B (ab16917, Abcam), normal rabbit IgG (CS200581, Millipore) were used for ChIP assay. DNA samples were acquired and then quantified by real-time PCR. Table 3 listed the primers.

**Table 3:** The primer sequences for ChIP-qPCR

Primers	Sequences	
*BMP2*	F	CGTCTAGTATTTTGGCATAGCATAGACG
	R	ACTCAATTTCCAGCCTGCTGTTT
*OSX*	F	AAGATGAAAGGGGCCGAAGG
	R	AATCCTCCAGCGGTGTTCAG
*OCN*	F	GCTGGGATGTTCTGTACCGT
	R	CCCTTCCCTGTGTCCTTAGC

### Animals

The animal studies had approval from the Medical Ethics Committee of West China Hospital of Stomatology, Sichuan University (WCHSIRB-D-2021-321). Embryos and newborn mice at embryonic day 17.5, postnatal day 0 and 3 (E17.5, P0 and P3) were obtained by time-mated pregnant C57BL/6 mice (Chengdu Dossy Experimental animals Co., Ltd.). After mice were euthanized, dental papilla and enamel organ tissues of mandibular first molars were separated under transmitted light microscope.

Five-weeks-old male Sprague Dawley rats (Chengdu Dossy Experimental animals Co., Ltd.) were used for pulpotomy. Rats were randomly separated into six groups (5 rats for per group), including group without pulpotomy (Control) and capping groups: Vitapex (Morita, Japan), lentivirus-scramble (GeneCopoeia), KDM6B-overexpression (pEZ-Lv105 lentivirus vector, GeneCopoeia), AAV-scramble (5′-CGCTGAGTACTTCGAAATGTC-3′, Genechemand AAV-miR-93-5p inhibitor (5′-ACCGCTACCTGCACGAACAGCACTTTGTTTTT-3′, GV479 vector, Genechem). Rats were anesthetized and the cavities on occlusal surfaces of maxillary first molars were prepared by 1/4-inch burs under water cooling. The dentin debris on pulp wound was flushed away by sterile saline. After the pulp surface were cleaned and covered by fresh blood, aseptic cotton pellet soaked in sterile saline was pressured on the pulp surface to stop bleeding. After the hemorrhage was under control, gelatin sponges were used to deliver capping agents. The cavities were protected with a thin layer of glass-ionomer cement and closely restored by composite resin at last. After 2 and 4 weeks, all rats were euthanized and the maxillae were fixed in 4% paraformaldehyde. For observing enhanced green fluorescent protein, the samples were embedded by Tissue-Tek O.C.T. Compound (Sakura). The tissue sections (6 μm) were obtained and photographed.

### MicroCT

The rats’ maxillae were collected for microCT analysis before decalcification. Tertiary dentin was analyzed by micro-CT scanner (μCT50, SCANCO MEDICAL AG) in a scanning resolution of 8 μm pixel size under the following settings: 70 kVp, 200 μA, AL 0.5 mm, 1 × 300 ms.

### Histologic and immunologic staining

The decalcified samples were embedded by paraffin for cutting into slices. Hematoxylin and eosin (Beyotime) staining was performed according to the instruction. For immunohistochemistry staining, the antibodies were BMP2 (AF5163, 1:200, Affinity Biosciences). For immunofluorescence staining, the antibodies were H3K27me3 (9733, 1:200, Cell Signaling Technology), KDM6B (ab169197, 1:250, abcam) and FITC conjugated secondary antibodies (1:400, Santa Cruz Biotechnology). Images were acquired by microscopy (Olympus, Japan).

### Statistical analysis

Relative mRNA levels were normalized with GAPDH*.* Relative microRNA levels were normalized with U6. Numerical data were presented as mean ± SD. GraphPad Prism 7 was used for data analysis. Student’s *t* test or ANOVA followed by Tukey’s test was used to evaluate statistical significance. *P* values < 0.05 were considered statistically significant.

## Results

### MicroRNA-93-5p downregulation is paralleled with KDM6B upregulation in tooth development and odontogenic differentiation of DPSCs

In our previous study [32], the expression of miRNAs from human tooth germ in bell stages were analyzed by miRNA microarray. The differentially expressed miRNAs were listed in the heat map (Additional file 1: Fig. S1A), suggesting that miR-93-5p of human tooth germ was significantly decreased during bell stage. Previous study confirmed that specific demethylase KDM6B of H3K27me3 marks was a key epigenetic regulator during dental papilla development and dynamically expressed in a spatiotemporal pattern [29]. For further investigated the epi-miRNAs interact with histone modification in dentin formation, we then searched the miRNA databases including TargetScanHuman7.2, miRbase Target and miRDB for miRNAs that not only differentially express during the bell stage, but also probably target on KDM6B. Afterwards, miR-93-5p was predicted to target with KDM6B (Additional file 1: Fig. S1B).

**Fig. 1:**
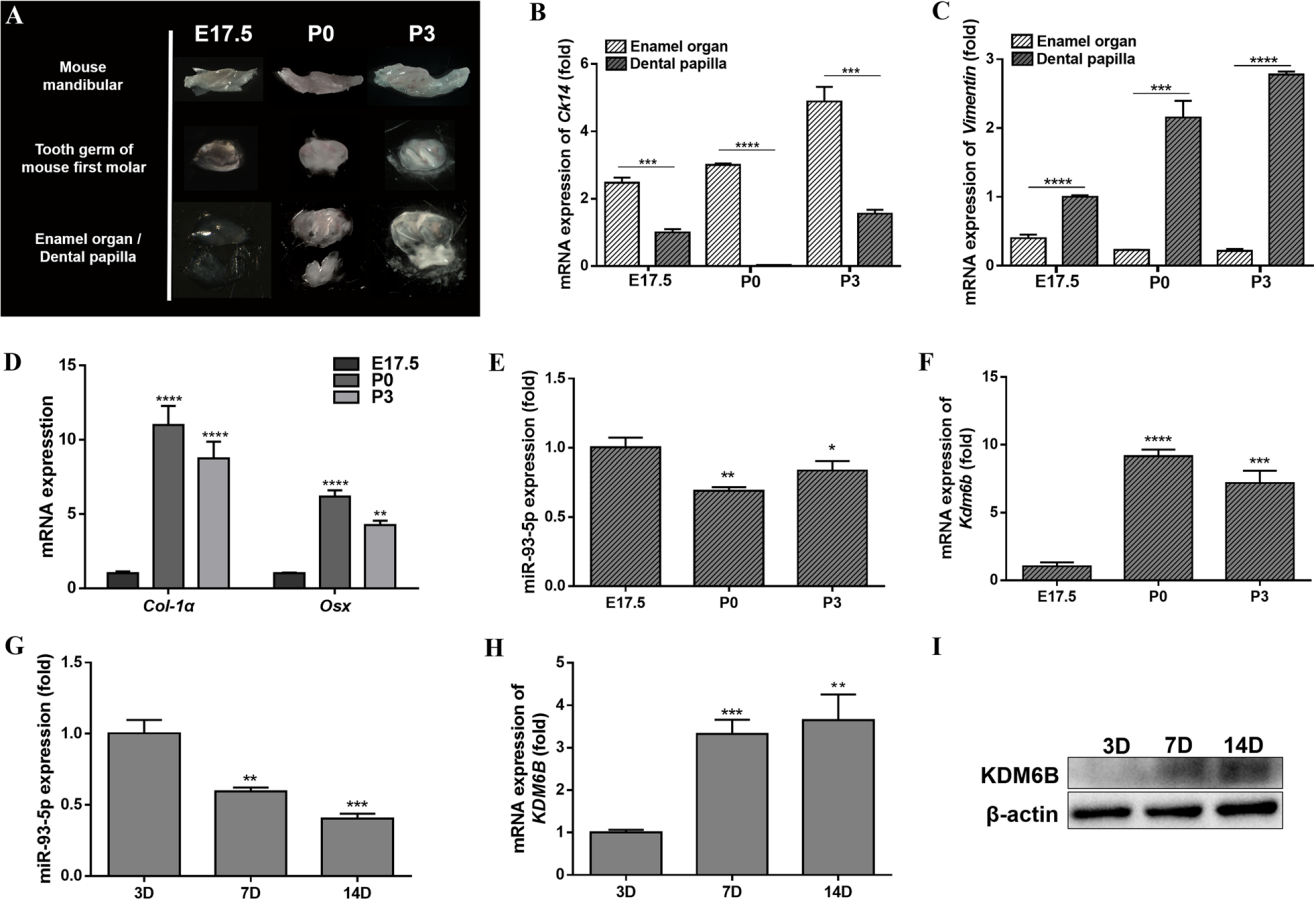
The expression pattern of miR-93-5p and KDM6B in tooth development and odontogenic differentiation of hDPSCs. **A** Dental papilla and enamel organ tissues of the first molar of mouse dissected under transmitted light microscope. **B**, **C** The expression levels of Ck14 and Vimentin in dissected enamel organ and dental papilla tissues of mouse, respectively. **D** The expression levels of Col-1α and Osx during the bell stage in mouse dental papilla tissues. **E**, **F** The expression levels of miR-93-5p and Kdm6b during the bell stage of mouse dental papilla, respectively. **G** The expression level of miR-93-5p during the odontogenic differentiation of hDPSCs. **H**, **I** The expression of KDM6B in hDPSCs during odontogenic induction at mRNA and proteins levels, respectively. *P < 0.05, **P < 0.01, ***P < 0.001 and ****P < 0.0001

Moreover, the dynamic expression trend of miR-93-5p in mice tooth germ from early to late bell stages was investigated. Dental papilla from mice first molar germs of embryonic 17.5 days (E17.5), postnatal 0 and 3 days (P0, P3) were separated under light microscope (Fig. 1A). The dental epithelial organ tissues expressed specific epithelial marker cytokeratin 14 (*Ck14*) while the dental papilla tissues significantly expressed specific mesenchymal marker *Vimentin* (Fig. 1B, C). In developing mouse dental papilla tissues, the expression of odontogenic genes collagen type-1α (*Col-1α*) and osterix (*Osx*) were upregulated from E17.5 to P3 (Fig. 1D). Along with the odontogenic differentiation process of mice dental papilla mesenchymal cells, miR-93-5p was down regulated (Fig. 1E) while *Kdm6b* showed an up-regulated trend (Fig. 1F).

To further delineate the expression pattern of KDM6B and miR-93-5p in odontogenic differentiation of adult dental mesenchymal cells, hDPSCs were cultured under odontogenic condition. MiR-93-5p was down-regulated when hDPSCs differentiated into odontoblasts (Fig. 1G). Furthermore, the expression of *KDM6B* was up-regulated in both mRNA and protein levels (Fig. 1H, I).

### MicroRNA-93-5p regulates odontogenic differentiation of hDPSCs

**Fig. 2:**
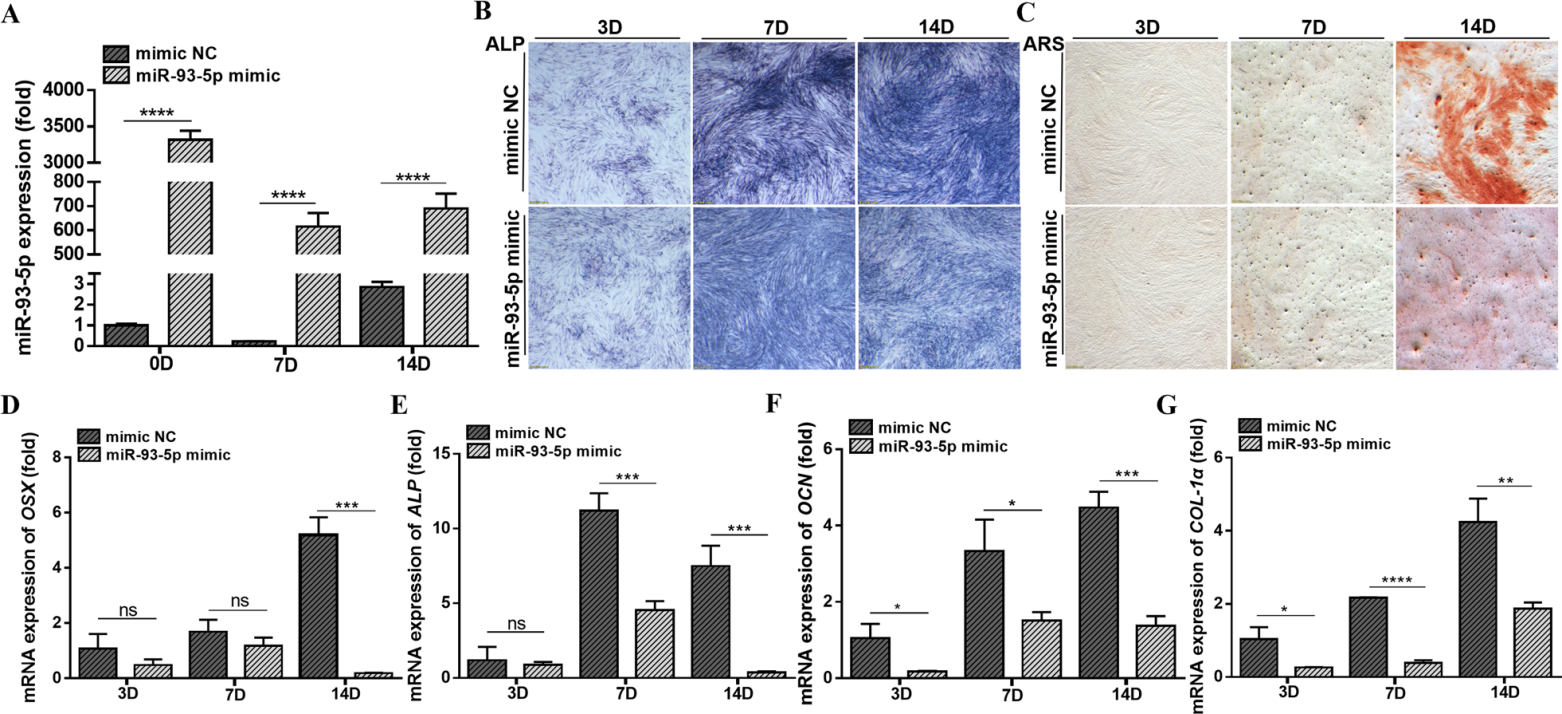
Upregulation of miR-93-5p suppresses the odontogenic differentiation of hDPSCs. **A** The miR-93-5p mimic was effectively transfected into hDPSCs. **B** MiR-93-5p mimic suppressed the ALP activity of hDPSCs during odontogenic differentiation. **C** MiR-93-5p mimic suppressed the mineralized nodule formation during the odontogenic differentiation of hDPSCs. **D**–**G** MiR-93-5p mimic decreased the mineralization indicators genes *OSX*, *ALP*, *OCN* and *COL-1α* when hDPSCs differentiated into odontoblasts. ns, not significant. *P < 0.05, **P < 0.01, ***P < 0.001 and ****P < 0.0001

**Fig. 3:**
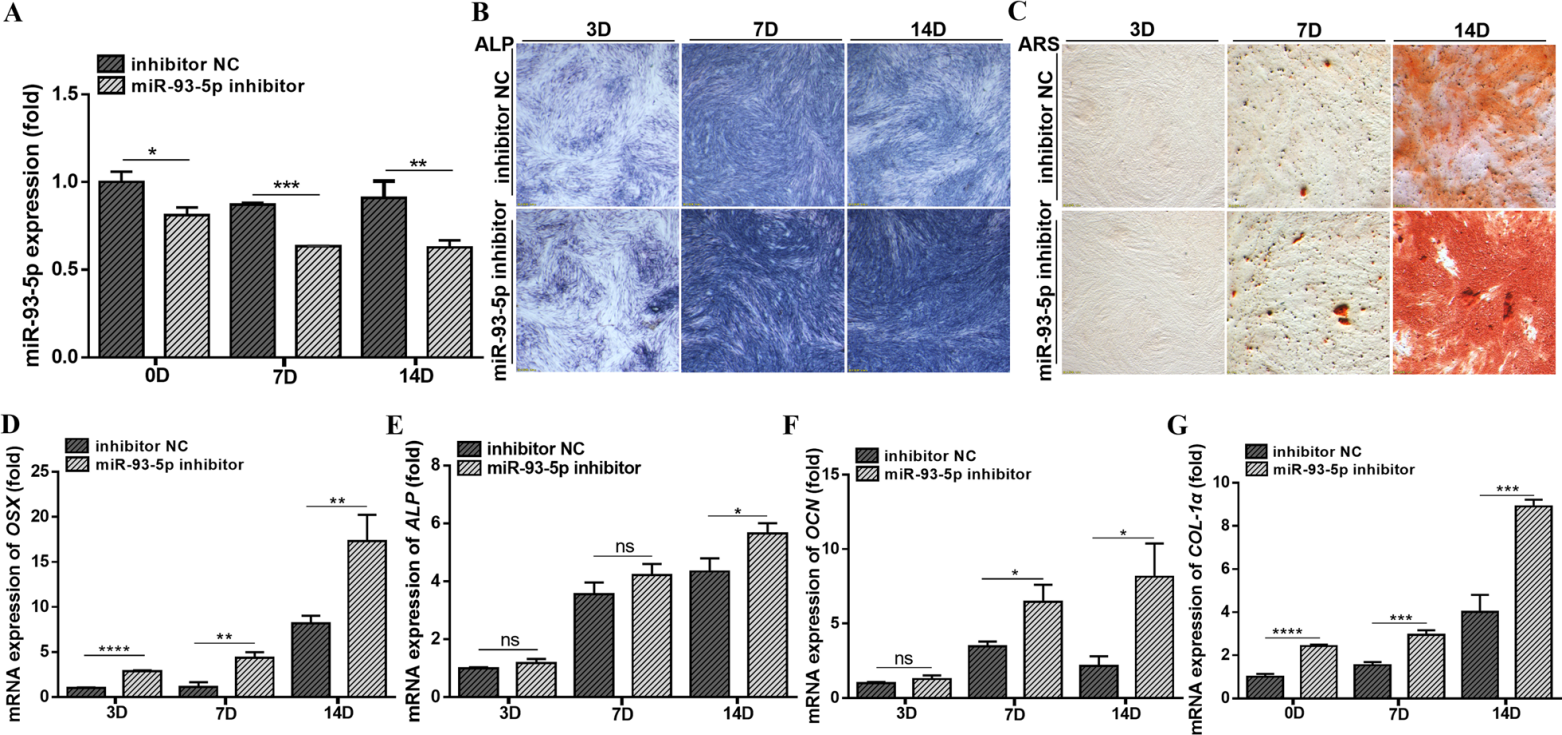
Downregulation of miR-93-5p promotes the odontogenic differentiation of hDPSCs. **A** The miR-93-5p inhibitor was effectively transfected into hDPSCs. **B** MiR-93-5p inhibitor increased the ALP activity of hDPSCs during odontogenic differentiation. **C** MiR-93-5p inhibitor induced mineralized nodule formation during the odontogenic differentiation of hDPSCs. **D**–**G** MiR-93-5p inhibitor increased the mineralization indicators genes *OSX*, *ALP*, *OCN* and *COL-1α* when hDPSCs differentiated into odontoblasts. ns, not significant. *P < 0.05, **P < 0.01, ***P < 0.001 and ****P < 0.0001

MicroRNA-93-5p mimic/inhibitor was transfected into hDPSCs effectively (Figs. 2A, 3A). After miR-93-5p mimic transfection and odontogenic induction, the activity of ALP was significantly suppressed while the mineralized nodule was reduced in hDPSCs (Fig. 2B, C). The mRNA expression of odontogenic genes *OSX*, *ALP*, osteocalcin (*OCN*) and *COL-1α* were significantly suppressed (Fig. 2D–G). In contrast, miR-93-5p inhibitor treatment promoted the ALP activity and mineralized nodule formation in hDPSCs, accordingly, mineralization indicators above were upregulated (Fig. 3B–G). Above data together indicating the miR-93-5p functionally regulating the odontogenic differentiation of hDPSCs.

### MicroRNA-93-5p targets KDM6B and influences H3K27me3 marks of BMP2

**Fig. 4:**
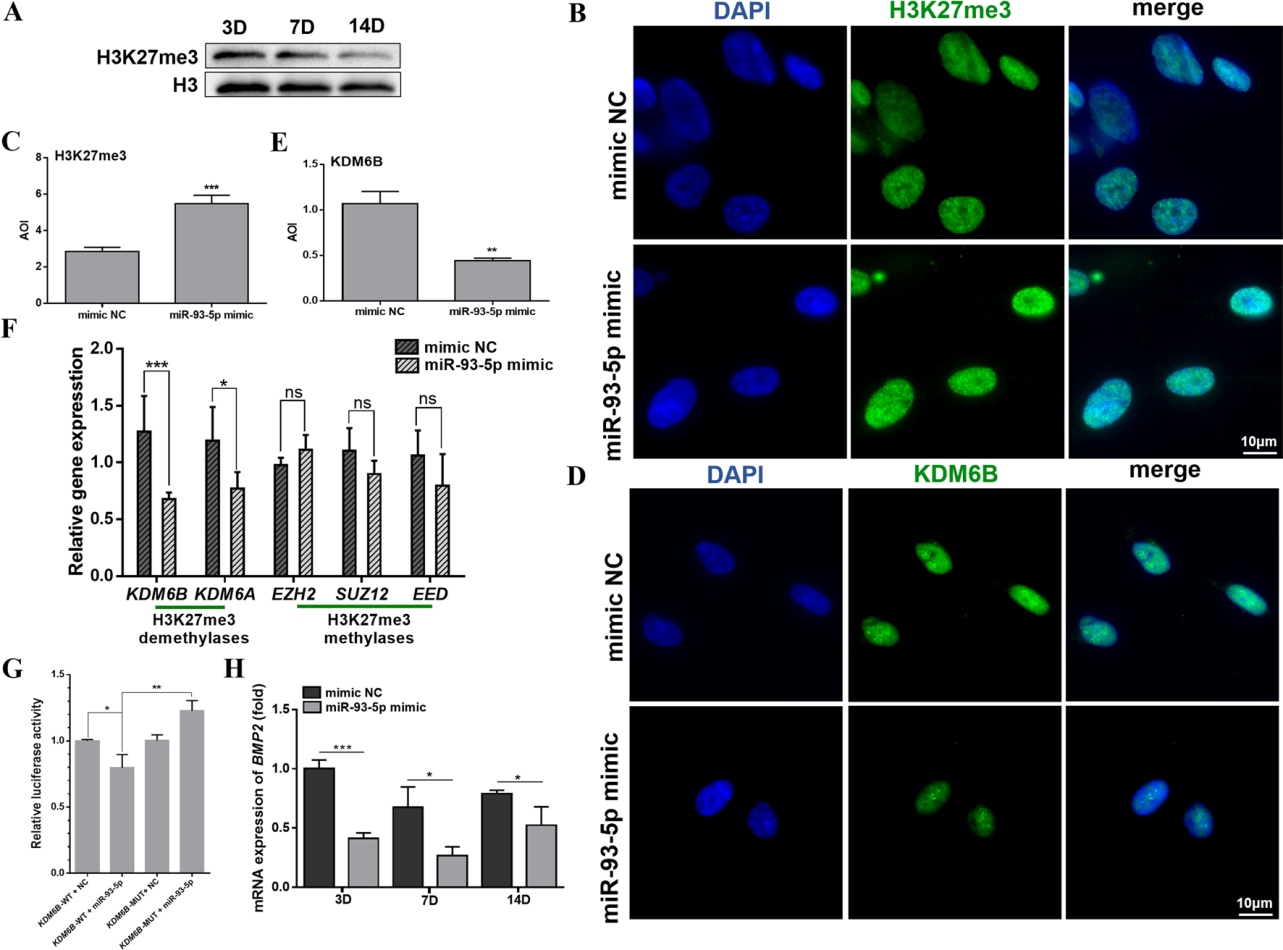
MiR-93-5p targets on KDM6B and influences H3K27me3 marks. **A** The H3K27me3 mark was down-regulated during the odontogenic differentiation of hDPSCs. **B**–**E** MiR-93-5p mimic decreased KDM6B and induced H3K27me3 marks during the odontogenic differentiation of hDPSCs. **F** After miR-93-5p mimic treatment, H3K27me3 demethylases were suppressed while H3K27me3 methylases were no significantly different. **G** Dual-luciferase assay confirmed that miR-93-5p targeted on the 3’ UTR of KDM6B. **H** MiR-93-5p mimic treatment down-regulated the expression level of *BMP2* in hDPSCs after odontogenic induction. ns, not significant. *P < 0.05, **P < 0.01 and ***P < 0.001

**Fig. 5:**
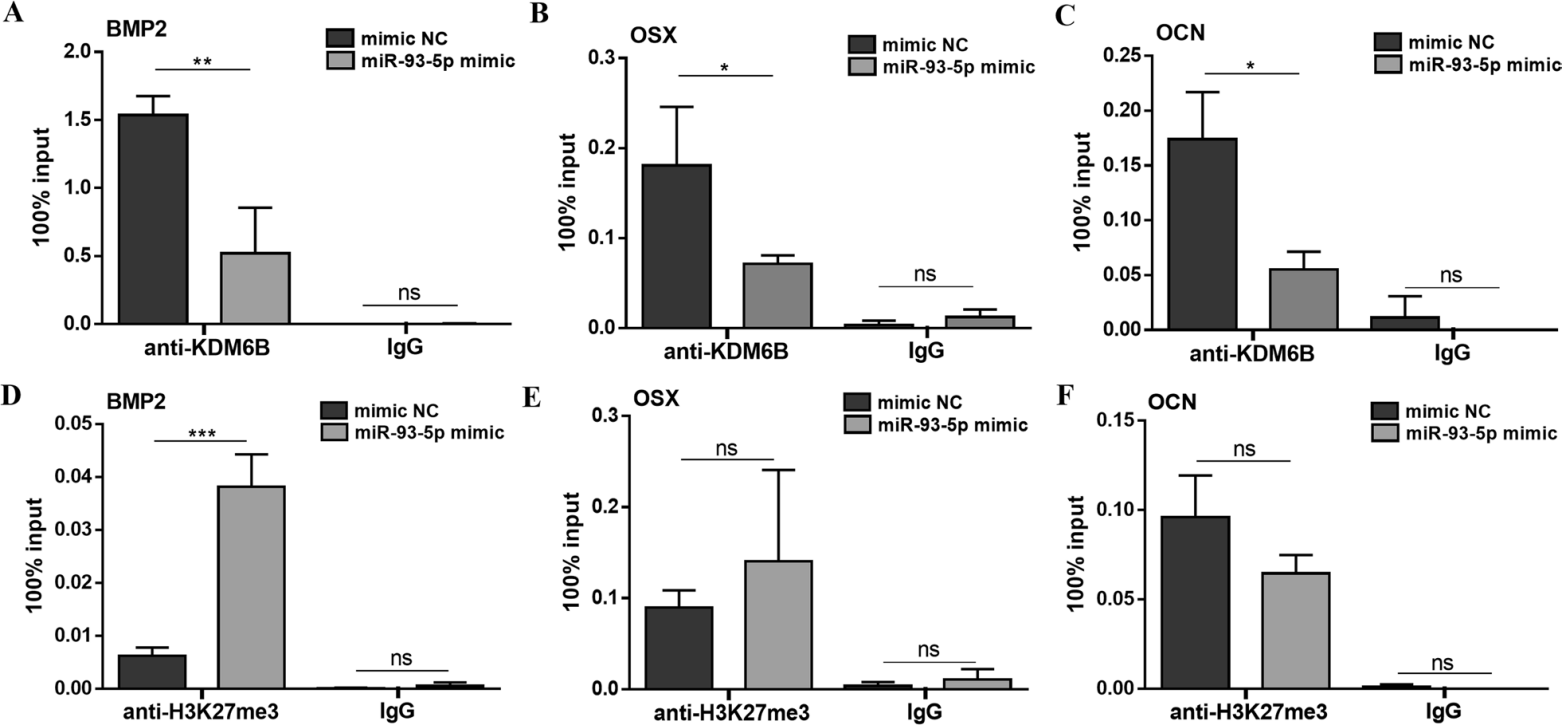
MiR-93-5p influences the KDM6B and H3K27me3 affinity on *BMP2* gene. **A**–**C** MiR-93-5p mimic treatment reduced the KDM6B affinity in promoter regions of *BMP2*, *OSX* and *OCN*, respectively. **D** MiR-93-5p mimic treatment increased the H3K27me3 marks in *BMP2* promoter regions. **E**, **F** MiR-93-5p mimic treatment had no significant effects on H3K27me3 marks in promoter regions of *OSX* and *OCN*. ns, not significant. *P < 0.05, **P < 0.01 and ***P < 0.001

During the odontogenic differentiation of hDPSCs, the H3K27me3 marks was down-regulated (Fig. 4A). After miR-93-5p mimic treatment, H3K27me3 marks in hDPSCs were significantly enriched (Fig. 4B, C). The KDM6B was targeted by miR-93-5p and down-regulated, the bonding site on KDM6B was also validated (Fig. 4D–G). The H3K27me3 methylases including EZH2, SUZ12, and EED were no different expression after miR-93-5p mimic treatment (Fig. 4F). BMP2 was further detected to be down-regulated in hDPSCs after miR-93-5p mimic transfected (Fig. 4H). To examined how miR-93-5p functioned on the *BMP2* transcription, ChIP-qPCR assays were conducted. After odontogenic induction for 7 days, the KDM6B affinity on *BMP2* promoters was decreased (Fig. 5A). Accordingly, increased H3K27me3 marks on *BMP2* promoters mirrored the loss of KDM6B occupancy (Fig. 5D). The different levels of KDM6B affinities on promoter regions of *OSX* and *OCN* had no significant effects on H3K27me3 marks (Fig. 3B, C, E, F). Above results suggested that miR-93-5p could influence H3K27me3 marks in *BMP2* promoter regions by targeting KDM6B, therefore epigenetically regulated the odontogenic differentiation of hDPSCs.

### MicroRNA-93-5p inhibitor induces dentin formation in rat pulpotomy model

**Fig. 6:**
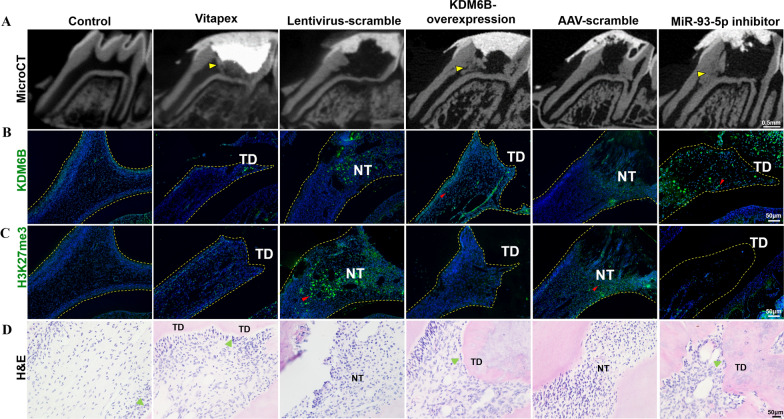
MiR-93-5p inhibitor promotes dentin formation in pulpotomy model. **A** MicroCT showed that KDM6B-overexpression and miR-93-5p inhibitor induced tertiary dentin formation in rat pulpotomy model after 4 weeks treatment (yellow arrows: tertiary dentin). **B**, **C** Immunofluorescence staining showed that KDM6B was upregulated while H3K27me3 marks were downregulated after miR-93-5p inhibitor or KDM6B-overexpression treatment (TD: tertiary dentin, NT: necrosis tissues, red arrows: positive cells). **D** H&E staining illustrated the tertiary dentin over the opening of tooth root canals (green arrows: odontoblasts)

**Fig. 7:**
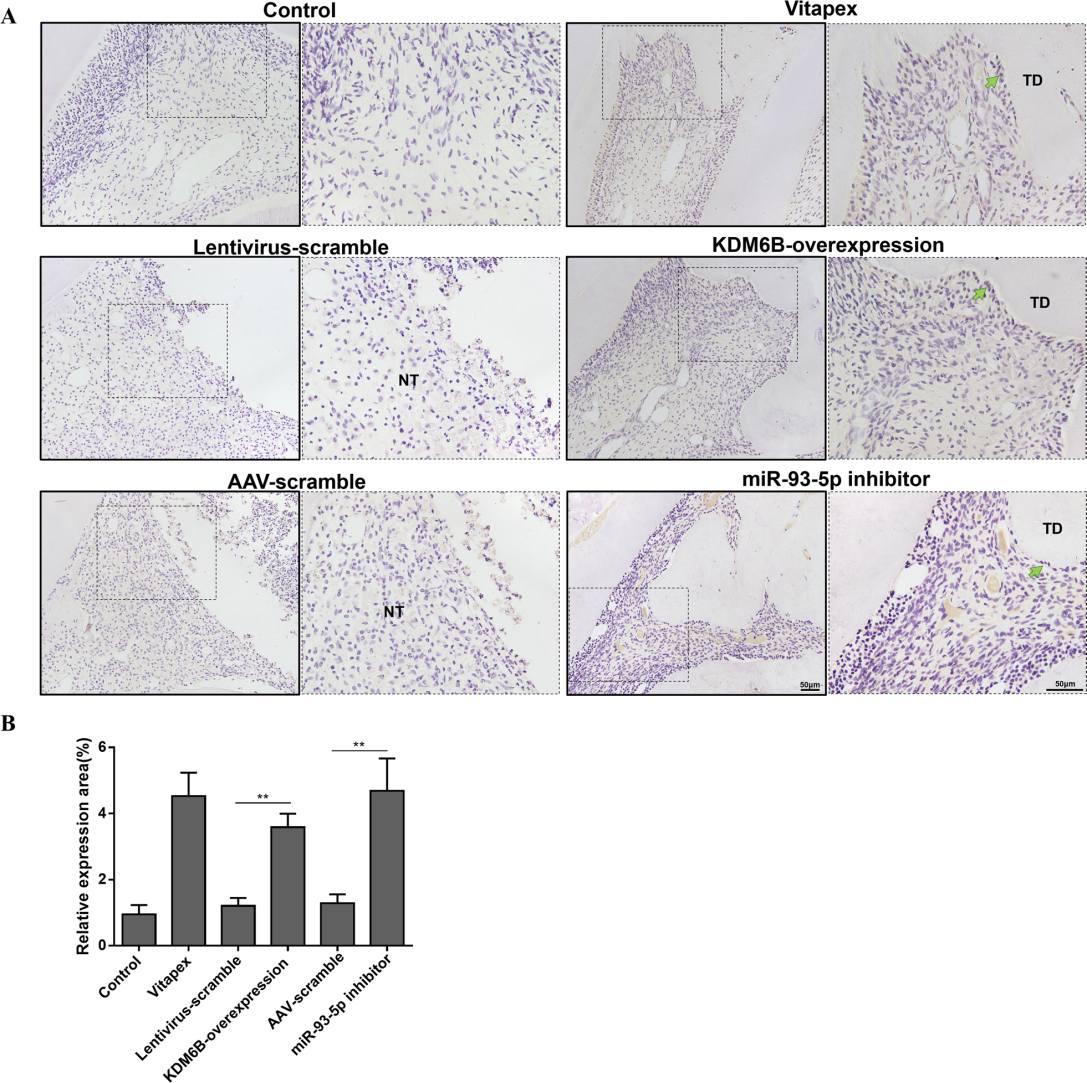
MiR-93-5p inhibitor upregulates the expression of BMP2 in pulp tissues. **A** Immunohistochemical staining of BMP2 (TD: tertiary dentin, NT: necrosis tissues, green arrows: positive cells). **B** Quantitative analysis of BMP2 positive area for each group. **P < 0.01

The pulpotomy was performed on rats’ maxillary first molars and the pulp cutting surfaces were capped by gelatin sponges with agents (Additional file 1: Fig. S2A–H). Fluorescence observation revealed that the capping agents with lentivirus and AAV vector were successfully transfected into the residual pulp of rats’ molars (Additional file 1: Fig. S2I). For MicroCT analysis, KDM6B-overexpression and miR-93-5p inhibitor treatment effectively promoted the formation of dentin bridges over the opening of tooth root canals after 4 weeks (Fig. 6A). In rat’s dental pulp, KDM6B-overexpression and miR-93-5p inhibitor treatment upregulated KDM6B accompanying with the downregulation of H3K27me3 marks, which is accordance with results in cultured hDPSCs (Fig. 6B, C). H&E staining showed that the necrotic pulp without tertiary dentin formation was in the lentivirus-scramble and AAV-scramble groups, while the KDM6B*-*overexpression and miR-93-5p inhibitor treatment induced the tertiary dentin formation above the pulp surfaces and protected the residual pulp tissues from inflammation (Fig. 6D). Accordingly, KDM6B-overexpression and miR-93-5p inhibitor treatment upregulated the expression of BMP2 in residual pulp tissues (Fig. 7).

## Discussion

MicroRNAs play an important role in organ development and pathological changes, not only via directly targeting on gene mRNAs, but also via their complicate interactions with other epigenetic factors. Some miRNAs work as epi-miRNAs to create controlled feedbacks by interacting with DNA methylation or histone modification marks. The epi-miRNAs related to the differentiation and proliferation of embryonic pluripotent stem cells are highly valued as potential molecular drugs for disease management and tissue regeneration [39].

As small molecular epigenetic factors, miRNAs have been confirmed to regulate the multiple signaling molecules underlying the whole process of odontogenesis by targeting various genes especially associated with cell differentiation [20, 40]. During the odontogenesis, the miR-34a can indirectly regulate the expression of ALP and promote odontogenic differentiation of dental apical papilla cells by inhibiting the Notch pathway [32, 41]. The miRNA-27 and miR-338-3p can promote odontoblast differentiation by activating Wnt/β-catenin signaling and directly suppress RUNX2 [42, 43]. As an epi-miRNA, miR-720 can suppress NANOG by DNMT3A and DNMT3B, accordingly regulate the proliferation and odontogenic differentiation of DPSCs [44].

Trimethylated of H3K27 is a repressive epigenetic mark and is crucial for relevant genes expression during tooth development. Studies demonstrated that the specific demethylase KDM6B was able to activate the expression of odontogenesis-associated genes *OSX*, *OCN* and *BMP2* in dental mesenchymal stem cells by regulating H3K27me3 marks [28, 45]. Since the bell stage of tooth development is critical period for dentin formation, the key factors and epigenetic machinery involved in this process should be well studied for exploring innovative therapies for dentin generation. In our previous study, the marks of H3K27me3 changed in a spatiotemporal trend during bell stage of tooth development. Considering the complex and multi-level relationship between epigenetic factors, we further analyzed the miRNAs express in human tooth germ between early and late bell stages by microarray analysis. After quering the miRNA databases (TargetScanHuman7.2, miRbase Target and miRDB), miR-93-5p was identified as the only candidate miRNA differentially expressed during the process of dentin formation and targeted to KDM6B. In addition, other H3K27me3 methylases including EZH2, SUZ12, and EED was not the target gene of miR-93-5p. The expression of EZH2, SUZ12, and EED showed no significant difference after the treatment of miR-93-5p mimics and inhibitors. These results suggested that miR-93-5p influences H3K27me3 by targeting to KDM6B, but not H3K27me3 methylases.

In a study of acute kidney treatment, the regulatory axis of KDM6B/H3K27me3/TNF-α was confirmed and the targeting site of miR-93-5p on KDM6B was identified by dual-luciferase reporter assay [36]. However, the expression pattern and underlying interaction between miR-93-5p and demethylase KDM6B in odontogenesis especially in dentinogenesis have not been reported. In present study, up-regulation of miR-93-5p suppressed the odontogenic differentiation while inhibition of miR-93-5p promoted hDPSCs differentiation into odontoblasts. These results suggested that miR-93-5p can work as an epi-miRNA and effectively regulate the odontogenic differentiation of hDPSCs in a multi-level epigenetic mechanism.

The dentinogenesis and osteogenesis are analogous process of synthesizing the extracellular matrix for hard tissue formation and share similar mineralization genes of *OSX*, *OCN* and *BMP2*. Previous studies have confirmed that *KDM6B* depletion can suppress the expression of *OSX*, *OCN* and *BMP2*, as well as the secretion of mineral matrix [28, 45, 46]. These results were consistent with our present results. The ChIP-qPCR data showed miR-93-5p suppressed the specific recruitment of KDM6B to the promoter region of *BMP2*, and consequently inhibited BMP2 expression by influencing the H3K27me3 marks on promoter region. The H3K27me3 marks with affinities of KDM6B on the promoter regions of *OSX* and *OCN* showed no significant alteration after miR-93-5p mimic treatment, suggesting the existence of complex and finer mechanisms underlying the regulation of *OSX* and *OCN* to maximize the benefit in varied tissue microenvironments. As reported, RUNX2 and OSX are early stages markers of osteo/odontoblastic differentiation, however, OCN mainly occurs late [47]. Studies have reported that in dental mesenchymal stem cells, KDM6B knockdown significantly altered the expression of downstream target gene DLX2 which is important for biomineralization by regulating the extracellular matrix proteins including OCN [28, 48]. After odontoblastic induction, the overexpression of lysine acetyltransferase p300 enriches H3K9ac mark on promoter regions and increase the expression of OCN [49]. During the odontogenic differentiation, OSX is in the downstream of IGF-I and MAPK signaling pathway in addition to the BMP-2/Smad/Runx2 axis [50, 51]. Besides, the suppressive epigenetic marks of H3K9me3 and H3K27me3 show a bivalent modification mode and locate predominantly on OSX during odontogenic differentiation of dental mesenchymal progenitors [52]. Additionally, under mineralized induction, the modification of active H3K4me3 marks on matrix-related genes *OCN*, *OSX*, *DMP1* and *DSPP* effectively promote odontogenic differentiation of hDPSCs [27]. All these studies provide further interpretations for the multiple regulatory mechanisms underlying the expression of *OSX* and *OCN*, explaining our relevant results to some extent.

Although microRNAs have been reported to function in the odontogenesis of hDPSCs through BMP2 pathway and subsequently regulating odontoblast markers DSP and DMP-1 [16], our study firstly proved miR-93-5p can work as an epi-miR by leading an innovative epigenetic network of BMP2 signals. As the BMP2 pathway severely influences the odontogenic differentiation of hDPSCs, miR-93-5p showed an effective impact on tertiary dentin formation by regulating KDM6B/H3K27me3/BMP2. In current study, we observed pulp capping agents that either elevated KDM6B expression or inhibited miR-93-5p significantly induced the formation of dentin bridge in rat pulpotomy model. Our results enriched the interaction between epigenetic factors, additionally, the underlying epigenetic regulation mechanism of miR-93-5p may be a prospective target to dentin regeneration and vital pulp therapy.

As a promising small biomolecular drug for pulp regeneration, the treatment effects of miRNAs are dependent on the mechanisms underlying hDPSCs proliferation, odontogenic differentiation, and inflammatory response [53, 54]. MiR-143-5p was reported to regulate the odontogenic differentiation by targeting MAPK14, and thus participated in the p38 MAPK signaling pathways [55]. Wnt1 was found to be a target of miR-140-5p, and the down-regulation of miR-140-5p promoted the odontogenic differentiation of DPSCs by activating Wnt1/β-catenin signaling pathway [56]. For inflamed human dental pulp cells stimulate by lipopolysaccharide, miR-146a and basic fibroblast growth factor worked cooperatively to promote the cell proliferation and odontogenic differentiation [57]. Besides, miRNAs also play a role in tissue defense and repair by regulating inflammation related genes. MiR-125a-3p has shown odonto-immunomodulatory properties by inhibiting NF-κΒ and TLR signaling [16]. Mesenchymal stem cell-derived exosomes miR-27b can inhibit sepsis by suppressing KDM6B and NF-κB signaling pathway [58]. Interestingly, miR-93-5p has also been proved to attenuate lipopolysaccharide-induced chondrocyte inflammation by targeting TLR4 and further inhibiting the NF-κB signaling [34]. The function of miR-93-5p in regulating inflammation also suggesting the miR-93-5p may have a potential advantage for vital pulp therapy.

## Conclusions

MiR-93-5p can target KDM6B and regulate H3K27me3 marks in the promoter region of *BMP2*, thus modulating the odontoblastic differentiation of hDPSCs and the formation of tertiary dentin. Our findings may not only advance our knowledge on the epigenetic regulation on the repair of pulp injury, but also provide a potential therapeutic measure to promote the success of vital pulp therapy and regenerative endodontics.

### Supplementary Information

**Additional file 1: Figure S1.** MiR-93-5p is downregulated in bell stage of human tooth germ and predicted to target on 3′UTR of KDM6B. (**A**) Heatmap of differentially expressed miRNAs during bell stage of human tooth germ (*P* < 0.05). (**B**) MiR-93-5p was predicted to target on KDM6B in databases of TargetScanHuman7.2, miRbase Target and miRDB. **Figure S2.** Rat pulpotomy model. (**A**–**H**) The pulpotomy on rats’ maxillary first molars. (**I**) The observation of green fluorescence protein in rats’ molars identified the transfection of agents was effective.

## Data Availability

The datasets used and/or analyzed during the current study are available from the corresponding author on reasonable request.

## Abbreviations

miRNAs: MicroRNAs
KDM6B: Lysine-specific demethylase 6B
H3K27me3: Lysine 27 trimethylation on histone 3
DPSCs: Dental pulp stem cells
hDPSCs: Human dental pulp stem cells
3′ UTR: 3′ Untranslated region
BMP: Bone morphogenetic protein
ALP: Alkaline phosphatase
ARS: Alizarin red S
OSX: Osterix
Col-1α: Collagen type I alpha
OCN: Osteocalcin
qRT-PCR: Quantitative reverse translation polymerase chain reaction
ChIP: Chromatin immunoprecipitation
AAV: Adreno-associated virus
FITC: Fluorescein isothiocyanate
