# Supplementary material for: MicroRNA-93-5p regulates odontogenic differentiation and dentin formation via KDM6B
Source: J Transl Med. 2024 Jan 13;22:54. doi: 10.1186/s12967-024-04862-z (PMC10787997; doi:10.1186/s12967-024-04862-z)
Supplement: Supplementary file 1 — Additional file 1: Figure S1. MiR-93-5p is downregulated in bell stage of human tooth germ and predicted to target on 3′UTR of KDM6B. (A) Heatmap of differentially expressed miRNAs during bell stage of human tooth germ (P < 0.05). (B) MiR-93-5p was predicted to target on KDM6B in databases of TargetScanHuman7.2, miRbase Target and miRDB. Figure S2. Rat pulpotomy model. (A–H) The pulpotomy on rats’ maxillary first molars. (I) The observation of green fluorescence protein in rats’ molars identified the transfection of agents was effective. [file 12967_2024_4862_MOESM1_ESM.docx]

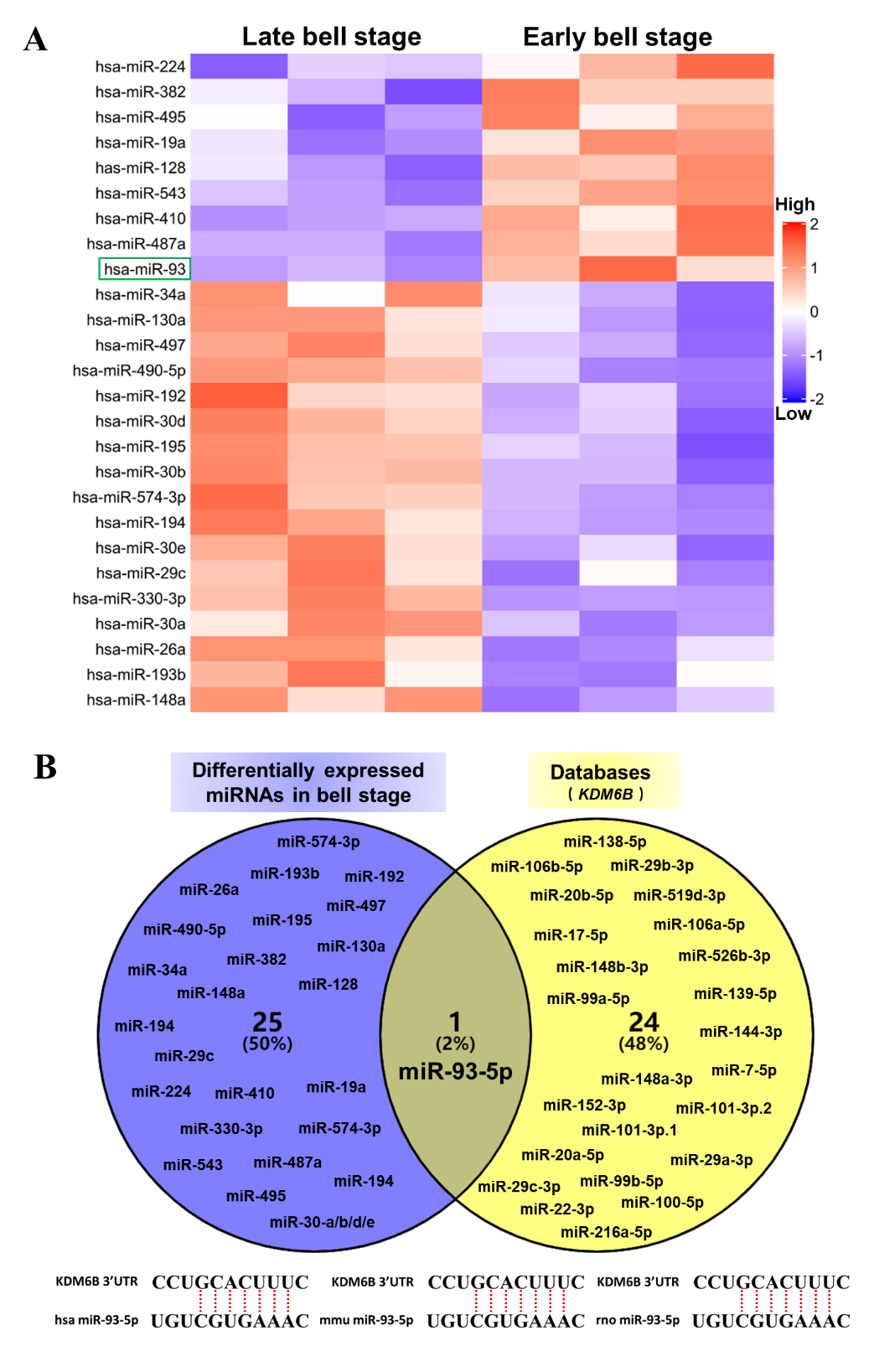


**Figure S1.** MiR-93-5p is downregulated in bell stage of human tooth germ and predicted to target on 3’UTR of KDM6B. (**A**) Heatmap of differentially expressed miRNAs during bell stage of human tooth germ (*P* ＜0.05). (**B**) MiR-93-5p was predicted to target on 3’UTR of KDM6B in databases of TargetScanHuman7.2, miRbase Target and miRDB.


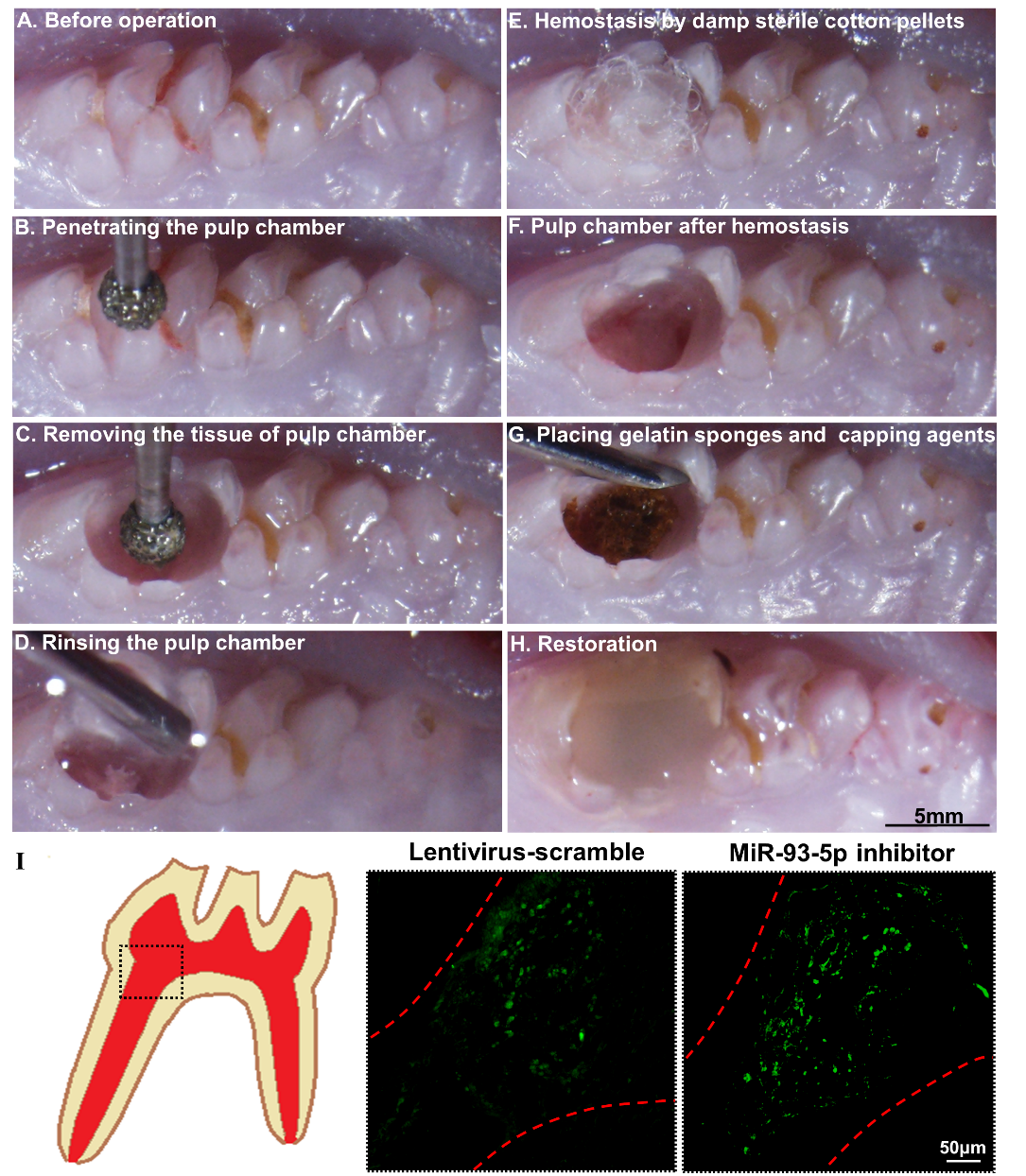


**Figure S2.** Rat pulpotomy model. (**A**-**H**) The pulpotomy on rats’ maxillary first molars. (**I**) The observation of green fluorescence protein in rats’ molars identified the transfection of agents was effective.
